# Efficacy and Safety of Combined Endovascular Embolization and Stereotactic Radiosurgery for Patients with Intracranial Arteriovenous Malformations: A Systematic Review and Meta-Analysis

**DOI:** 10.1155/2021/6686167

**Published:** 2021-04-14

**Authors:** Zhiqun Jiang, Xuezhi Zhang, Xichen Wan, Minjun Wei, Yue Liu, Cong Ding, Yilv Wan

**Affiliations:** Department of Neurosurgery, The First Affiliated Hospital of Nanchang University, Nanchang, China

## Abstract

Whether the use of endovascular embolization could provide additional benefits in patients treated with stereotactic radiosurgery (SRS) for intracranial arteriovenous malformations (IAVMs) remains controversial. The current meta-analysis was conducted to assess the efficacy and safety of SRS with and without prior endovascular embolization in patients with IAVMs. The electronic databases of PubMed, EmBase, and Cochrane Library were systematically searched for eligible studies published from inception to August 12, 2020. The pooled results for obliteration rate, rehemorrhage rate, and permanent neurological deficits were calculated by odds ratios (ORs) with 95% confidence intervals (CIs) using the random-effects model. The sensitivity analysis, subgroup analysis, and publication bias for investigated outcomes were also evaluated. Nineteen studies (two prospective and 17 retrospective studies) involving a total of 3,454 patients with IAVMs were selected for the final meta-analysis. We noted that prior embolization and SRS were associated with a lower obliteration rate compared with SRS alone (OR, 0.57; 95% CI, 0.44–0.74; *P* < 0.001). However, prior embolization and SRS were not associated with the risk of rehemorrhage (OR, 1.05; 95% CI, 0.81–1.34; *P* = 0.729) and permanent neurological deficits (OR, 0.80; 95% CI, 0.48–1.33; *P* = 0.385) compared with SRS alone. The sensitivity analysis suggested that prior embolization might reduce the risk of permanent neurological deficits in patients with IAVMs treated with SRS. The treatment effects of prior embolization in patients with IAVMs could be affected by nidus volume, margin dose, intervention, and follow-up duration. This study found that prior embolization was associated with a reduced risk of obliteration in patients with IAVMs treated with SRS. Moreover, prior embolization might reduce the risk of permanent neurological deficits in patients with IAVMs.

## 1. Introduction

Intracranial arteriovenous malformations (IAVMs) are congenital, heterogeneous, and rare vascular abnormalities that can cause intracranial hemorrhage, headache, seizure, and death [1]. IAVMs with an abnormal nidus of blood vessels shunt blood from the arterial to the venous system and bypass an intervening capillary bed [2]. These lesions account for 2–3% of symptomatic hemorrhages, and the hemorrhage rate was 2–4% annually when patients were left untreated [3, 4]. The primary treatment goal for IAVMs was to reduce rupture risk and ameliorate symptoms, and the spontaneous hemorrhage rate in IAVMs ranged from 2% to 5% [5, 6]. Moreover, IAVMs with hemorrhage had morbidity and mortality rates ranging from 53% to 81% and 10% to 18%, respectively [7, 8]. Presently, the standard treatment strategies for IAVMs included conventional microsurgical excision, stereotactic radiosurgery (SRS), endovascular embolization, and a combination of the abovementioned strategies according to the size and anatomic location, clinical presentation, and angioarchitecture of the IAVMs [9, 10].

Currently, the treatment effects of SRS were inversely related to the size of the malformation and treatment dose, which could provide more beneficial effects for IAVMs with size ≤ 3 cm. Studies have found that the obliteration rate at 3 years ranged from 55% to 81% in patients with IAVMs staged Spetzler-Martin 1 and 2 treated with 20–25 Gy [11–15], while the obliteration rate after 5 years of SRS in patients with large and more complex IAVMs was <50% [16–18]. Therefore, the risk of hemorrhage was not significantly reduced after 1–2 years of SRS prior to angiographic obliteration [19]. Therefore, endovascular embolization prior to SRS should introduce as a neurointerventional minimally invasive approach for patients with IAVMs. Although endovascular embolization rarely provided complete treatment for IAVMs, it could improve the natural history of patients at high risk of hemorrhage owing to intranidal or perinodal aneurysms and large venous varices [20–22]. However, whether the use of SRS following by prior embolization could provide additional benefits than SRS alone in patients with IAVMs was not determined. Therefore, the current systematic review and meta-analysis were conducted to obtain a comprehensive, quantitative evidence to compare the efficacy and safety of SRS following embolization with SRS alone in patients with IAVM new results.

## 2. Materials and Methods

### 2.1. Data Sources, Search Strategy, and Selection Criteria

The current systematic review and meta-analysis were conducted following the Preferred Reporting Items for Systematic Reviews and Meta-Analysis Protocol [23]. Studies that compared the efficacy and safety of prior embolization for patients with IAVMs treated with SRS were eligible for this study, and restriction was not placed on published language and status. The electronic databases of PubMed, EmBase, and Cochrane Library were systematically searched for eligible studies from their inception until August 12, 2020, and the following search terms were used: (“intracranial arteriovenous malformations” or “brain arteriovenous malformations” or “cerebral arteriovenous malformations”) and (“radiosurgery” or “stereotactic radiosurgery” or “radiotherapy” or “linear accelerator (LINAC)” or “Gamma Knife” or “CyberKnife”) and (“embolization” or “particles” or “N-butyl cyanoacrylate” or “Onyx”). Then, the reference lists of retrieved studies were reviewed manually to select any new study that met the inclusion criteria.

The literature search and study screening process were independently performed by two reviewers, and the disagreement between reviewers was resolved by discussion until a consensus was reached. The study was included if they met all the inclusion criteria: (1) patients, all patients diagnosed with IAVMs, irrespective of disease status; (2) intervention, SRS following embolization; (3) control, SRS alone; (4) outcomes, the study reported on obliteration rate, rehemorrhage rate, or permanent neurological deficits; and (5) study design, original article and unrestricted design type.

### 2.2. Data Collection and Quality Assessment

A standardized protocol guided the two reviewers to abstract the following items: first author's name, publication year, study design, country, sample size, male proportion, mean age of patients, hemorrhages proportion, nidus size, nidus volume, margin dose, intervention, follow-up duration, and reported outcomes. Moreover, the methodological quality of the individual study was independently assessed using the Newcastle-Ottawa Scale (NOS) by two reviewers, which was based on selection (4 items, 4 stars), comparability (1 item, 2 stars), and outcome (3 items, 3 stars), and “staring system” for each study ranged from 0 to 9 [24]. Any inconsistency between the two reviewers for data abstracted and quality assessment was resolved by an additional reviewer referring to the full text of the original article.

### 2.3. Statistical Analysis

The results of reported outcomes were assigned as categorical data, and odds ratio (OR) with 95% confidence interval (CI) was calculated from the event and sample size in each group of each study. Subsequently, the pooled effect estimates were calculated using the random-effects model, which could consider the underlying variations across included studies [25, 26]. Heterogeneity among included studies for each outcome was assessed by *I*^2^ and *Q* statistic, and significant heterogeneity was defined as *I*^2^ > 50.0% or *P* value for *Q* statistic < 0.10 [27, 28]. The robustness of pooled conclusion was assessed using the sensitivity analysis through sequential exclusion of individual study [29]. Subgroup analyses for obliteration rate, rehemorrhage rate, and permanent neurological deficits were also performed based on the study design, country, sample size, mean age, nidus volume, margin dose, intervention, follow-up, and study quality, and difference between subgroups was assessed using the interaction *P* test, which assumed that the distribution of effect estimate met the normality [30]. The funnel plot, Egger, and Begg tests were used to assess the potential publication bias [31, 32]. The trim-and-fill method was applied to adjust for potential publication bias if significant publication bias was detected [33]. All statistical analyses in this study were conducted using the Stata software (version 10.0; StataCorp, Texas, USA).

## 3. Results and Discussion

### 3.1. Literature Search

**Figure 1:**
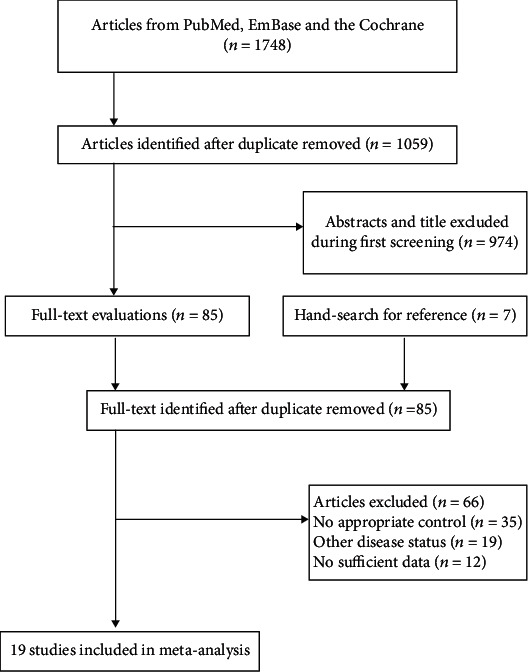
Flow diagram of the literature search and study selection process.

The initial electronic search yielded 1,748 articles, and 689 were excluded owing to duplicate titles. Then, 974 of 1,059 studies were excluded because of unrelated topics. The remaining 85 studies were retrieved for further full-text evaluations, and 66 studies were excluded owing to inappropriate control (*n* = 35), other disease statuses (*n* = 19), and insufficient data (*n* = 12). Then, a review of the reference lists of the remaining 19 studies found seven potentially included studies; then, these studies were excluded because of inappropriate control and insufficient data, which were noted in the 66 excluded studies by full-text evaluations. Finally, 19 studies were selected for the final meta-analysis [34–52]. The details regarding the literature search and study selection process are presented in Figure 1.

### 3.2. Study Characteristics

Of the 19 included studies, two studies had a prospective design [40, 44], and the remaining 17 studies had a retrospective design [34–39, 41–43, 45–52]. The baseline characteristics of the included studies and patients are summarized in Table 1. The included studies recruited a total of 3,454 patients with IAVMs, and the sample size ranged from 22 to 944. Six studies were conducted in Eastern countries [34, 38, 39, 42, 45, 52], and the remaining 13 studies were conducted in Western countries [35–37, 40, 41, 43, 44, 46–51]. Five studies applied linear accelerator radiosurgery as SRS [34–36, 41, 48], nine studies used Gamma Knife surgery as SRS [37, 38, 42, 43, 45, 46, 49, 50, 52], and the remaining five studies applied combined strategies as SRS [39, 40, 44, 47, 51]. The follow-up duration ranged from 24.0 to 180.0 months. The quality of included studies was assessed using the NOS: six studies had 8 stars [36, 42, 46, 48–50], six studies had 7 stars [35, 40, 43, 45, 51, 52], and the remaining seven studies had 6 stars [34, 37–39, 41, 44, 47].

### 3.3. Obliteration Rate

**Figure 2:**
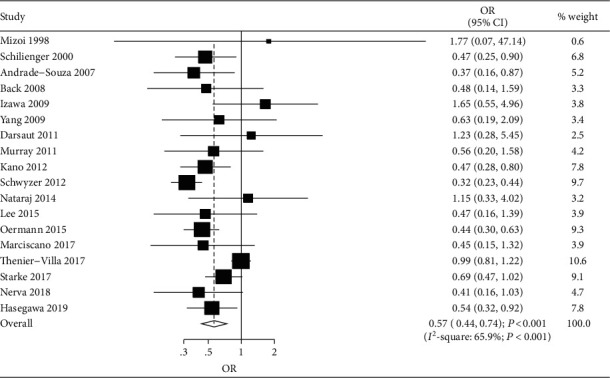
Forest plot of SRS following embolization versus SRS alone on the risk of obliteration rate.

A total of 18 studies reported the effects of SRS following embolization versus SRS alone on the obliteration rate [34–50, 52]. We noted that SRS following embolization was associated with a lower obliteration rate compared with SRS alone (OR, 0.57; 95% CI, 0.44-0.74; *P* < 0.001; Figure 2), and significant heterogeneity was observed across included studies. The sensitivity analysis found that the pooled conclusion was not altered by sequential exclusion of individual study (Supplement 1). Although the subgroup analyses found a significant difference in the obliteration rate in most subgroups between SRS following embolization and SRS alone, we noted that SRS following embolization was not associated with the risk of obliteration in prospective pooled studies, studies that did not report on SRS strategy, or studies with low quality (Table 2). Moreover, the treatment effect between SRS following embolization and SRS alone on the risk of obliteration could be affected by nidus volume (*P* < 0.001), margin dose (*P* < 0.001), intervention (*P* < 0.001), and follow-up duration (*P* = 0.036). Finally, although the Egger test suggested no significant publication bias for the obliteration rate (*P* = 0.472), the Begg test suggested potential significant publication bias for the obliteration rate (*P* = 0.028) (Supplement 2). The conclusion was not changed after adjusting for publication bias using the trim-and-fill method [33].

No: number; yrs: years; vol: volume; NA: not available; SM: Spetzler-Martin; Retro: retrospective; Pro: prospective.

### 3.4. Rehemorrhage

**Figure 3:**
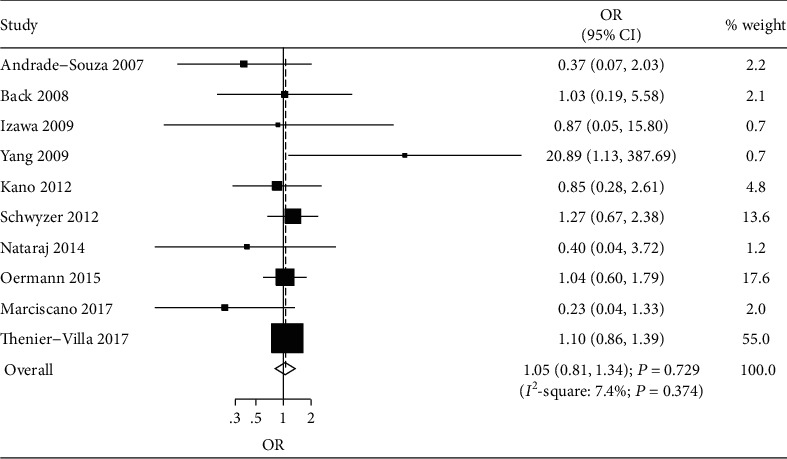
Forest plot of SRS following embolization versus SRS alone on the risk of rehemorrhage.

A total of 10 studies reported the effects of SRS following embolization versus SRS alone on the risk of rehemorrhage [36–39, 42–44, 46–48]. We noted that SRS following embolization was not associated with the risk of rehemorrhage compared with SRS alone (OR, 1.05; 95% CI, 0.81–1.34; *P* = 0.729; Figure 3), and unimportant heterogeneity was detected across included studies. This conclusion showed stability through sequential exclusion of individual study (Supplement 1). There was no significant difference in the risk of rehemorrhage in all subgroups between SRS following embolization and SRS alone, and no predefined factors could affect the treatment effects (Table 2). There was no significant publication bias for rehemorrhage (*P* value for Egger test, 0.512; *P* value for Begg test, 0.721; Supplement 2).

### 3.5. Permanent Neurological Deficits

**Figure 4:**
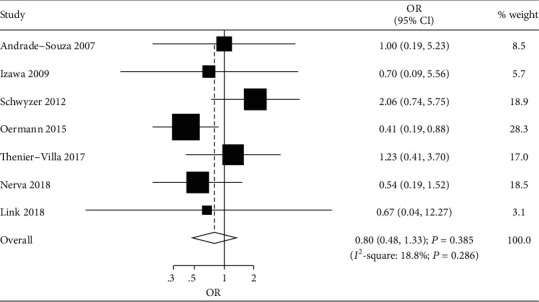
Forest plot of SRS following embolization versus SRS alone on the risk of permanent neurological deficits.

Seven studies reported the effects of SRS following embolization versus SRS alone on the risk of permanent neurological deficits [36, 38, 43, 46, 48, 50, 51]. The summary OR indicated no significant difference between SRS following embolization and SRS alone for the risk of permanent neurological deficits (OR, 0.80; 95% CI, 0.48–1.33; *P* = 0.385; Figure 4), and significant heterogeneity was noted among included studies. The sensitivity analysis indicated that SRS following embolization might reduce the risk of permanent neurological deficits than SRS alone after excluding the study conducted by Schwyzer et al. [43] (Supplement 1). The subgroup analysis indicated that SRS following embolization was associated with a reduced risk of permanent neurological deficits when the follow-up duration was <60.0 months (Table 2). No significant publication bias for permanent neurological deficits was observed (*P* value for Egger test: 0.614; *P* value for Begg test, 1.000; Supplement 2).

## 4. Discussions

This systematic review and meta-analysis were performed based on published articles and compared the treatment effects between SRS following embolization and SRS alone in patients with IAVMs. This study recruited 3,454 patients with IAVMs from two prospective and 17 retrospective studies across a broad range of patient characteristics. This study found that SRS following embolization was associated with a reduced risk of obliteration compared with SRS alone. Moreover, there were no significant differences between SRS following embolization and SRS alone for the risk of rehemorrhage and permanent neurological deficits. The sensitivity analysis found that SRS following embolization might play a protective role on the risk of permanent neurological deficits than SRS alone. Finally, the treatment effects between SRS following embolization and SRS in patients with IAVMs could be affected by nidus volume, margin dose, intervention, and follow-up duration.

Several systematic reviews and meta-analyses have been conducted to compare the treatment effects between SRS following embolization and SRS alone in patients with IAVMs. A review on 10 studies conducted by Xu et al. found that SRS following embolization was associated with a lower obliteration rate, while there were no significant effects on the risk of rehemorrhage and permanent neurological deficits [53]. However, this study provided pooled effect estimates for the treatment effects between SRS following embolization and SRS alone, and whether the treatment effects vary according to patient characteristics were not addressed. An updated meta-analysis conducted by Russell et al. included 12 studies and found that the combination of embolization and SRS was associated with lower obliteration rate compared with SRS alone, while other outcomes were not addressed, and the pooled effect estimates were not calculated [54]. Zhu et al. conducted a meta-analysis of six studies to compare the benefit and risk of Gamma Knife surgery after embolization in patients with residual IAVMs. They point out that Gamma Knife surgery following embolization could significantly reduce the obliteration rate, while it did not affect the risk of rehemorrhage and permanent neurological deficits [55]. However, this study focused on Gamma Knife surgery as an SRS strategy, while other types of SRS were not addressed. Moreover, the analysis only included six studies, and the power might be inadequate to detect potential differences between groups. Therefore, the current updated systematic review and meta-analysis were conducted to compare the treatment effects between SRS following embolization and SRS alone in patients with IAVMs.

The overall result of this study found that SRS following embolization was associated with a lower obliteration rate than SRS alone, which was consistent with the results of previous meta-analyses [53–55]. Several reasons could explain this pooled conclusion: (1) the radiation beams delivered by SRS could be absorbed or scattered by embolic agents and cause a reduced overall dose to the nidus [56], (2) embolization could convert the nidus from dormant status to a dynamic status by promoting angiogenesis within IAVMs [57], (3) the embolization in IAVMs could increase the difficulty to define the nidus by obscuring its boundaries and cause increased risk of SRS treatment failure [58], (4) embolization could fragment the nidus into noncontiguous compartments and increase the difficulty of SRS target [59], and (5) the embolized portions of IAVMs was not the target of SRS, which could recanalize at the post-SRS latency period and cause a patent nidus on follow-up neuroimaging [60]. Moreover, subgroup analyses found the treatment effects between SRS following embolization and SRS alone for the risk of obliteration could be affected by nidus volume, margin dose, intervention, and follow-up duration. Finally, we noted no significant differences between groups for the risk of obliteration in prospective studies, studies that did not report SRS strategy, or studies with low quality. These results could be explained by the statistical power, severity of nidus, intensity of intervention, and reliability of results in the individual study.

The pooled results found that SRS following embolization was not associated with the risk of rehemorrhage compared with SRS alone. Almost all included studies reported similar results. Moreover, the results showed stability and were not altered by using a sensitivity and subgroup analyses. This result could be explained by the difference in the nidus size and volume between the SRS following embolization and SRS alone groups. Furthermore, although SRS following embolization was not associated with the risk of permanent neurological deficits than SRS alone, the sensitivity analysis found that SRS following embolization might reduce the risk of permanent neurological deficits. In addition, the protective role of SRS following embolization on the risk of permanent neurological deficits was mainly observed in studies with follow-up duration of <60.0 months. The potential reason for this could be that most permanent neurological deficits mainly occurred in shorter follow-up duration after SRS.

Several limitations of this study should be acknowledged. First, most included studies (17/19) had a retrospective observational design, and the conclusions of this study were based on lower evidence level, which should be recommended cautiously. Second, the disease status and experience of the clinician are different across included studies, which could affect the prognosis of IAVMs. Third, the heterogeneity across included studies was not fully explained using sensitivity and subgroup analyses, which restricted the reliability of pooled conclusions. Fourth, the background treatment options and rehabilitation strategies were not addressed, which could affect the treatment effects between groups for the midterm and long-term outcomes. Finally, the inherent limitations of the meta-analysis based on published articles include publication bias and analysis based on pooled data.

## 5. Conclusions

This study found that SRS following embolization could reduce the risk of the obliteration rate than SRS alone. Moreover, the sensitivity analysis suggested that SRS following embolization might play a protective role on the risk of permanent neurological deficits. However, SRS following embolization was not associated with the risk of rehemorrhage. These conclusions should be verified in further large-scale randomized controlled trials.

## Figures and Tables

**Table 1:** Characteristics of included studies and patients.

Study	Study design	Country	Sample size (intervention/control)	Male (%)	Mean age (yrs)	Hemorrhages (%)	Nidus size (cm)	Nidus vol (ml)	Margin dose (Gy)	Intervention	Follow-up (months)	NOS score
Mizoi et al. 1998 [34]	Retro	Japan	32 (31/1)	NA	NA	NA	NA	10.9	19.2	Linear accelerator radiosurgery	45.7	6
Schilienger et al. 2000 [35]	Retro	France	169 (65/104)	62	33	NA	2.2	2.5	25.0	Linear accelerator radiosurgery	48-96	7
Andrade-Souza et al. 2007 [36]	Retro	Canada	94 (47/47)	NA	39	45.8	2.4	5.6	15.0	Linear accelerator radiosurgery	44	8
Back et al. 2008 [37]	Retro	USA	69 (15/54)	45	40	NA	NA	5.1	NA	Gamma knife surgery	36	6
Izawa et al. 2009 [38]	Retro	Japan	252 (15/237)	62	30	54.4	NA	5.0	20.0	Gamma knife surgery	81.5	6
Yang et al. 2009 [39]	Retro	Korea	46 (25/21)	59	32	37.0	NA	29.5	14.1	Linear accelerator and gamma knife	63.6	6
Darsaut et al. 2011 [40]	Pro	USA	42 (17/25)	NA	12	NA	NA	27.4	21.2	Charged particle radiation, linear accelerator, CyberKnife, or gamma knife	36	7
Murray 2011 [41]	Retro	USA	78 (57/21)	48	34	39.7	NA	17.7	18.1	Linear accelerator radiosurgery	34.8	6
Kano et al. 2012 [42]	Retro	China	240 (120/120)	50	33	NA	2.8	7.1	18.0	Gamma knife surgery	70.8	8
Schwyzer et al. 2012 [43]	Retro	USA	944 (215/729)	50	34	51.9	2.2	3.2	20.1	Gamma knife surgery	66.6	7
Nataraj et al. 2014 [44]	Pro	UK	54 (17/37)	54	41	NA	NA	NA	NA	Charged particle radiation, linear accelerator, CyberKnife, or gamma knife	24.0	6
Lee et al. 2015 [45]	Retro	China	75 (25/50)	40	41	NA	NA	3.2	20.7	Gamma knife surgery	25.2	7
Oermann et al. 2015 [46]	Retro	USA	484 (242/242)	42	31	50.5	2.6	4.3	20.0	Gamma knife surgery	54.6	8
Marciscano et al. 2017 [47]	Retro	USA	42 (22/20)	33	25	36.0	NA	13.1	15.4	Linear accelerator, CyberKnife, or gamma knife	114.0	6
Thenier-Villa et al. 2017 [48]	Retro	Spain	195 (47/148)	56	38	44.6	NA	NA	16.8	Linear accelerator radiosurgery	180.0	8
Starke et al. 2017 [49]	Retro	US and Canada	357 (78/279)	54	13	68.6	2.3	3.5	21.0	Gamma knife surgery	92.0	8
Nerva et al. 2018 [50]	Retro	USA	70 (20/50)	60	36	40.0	1.6	13.0	19.0	Gamma knife surgery	49.2	8
Link et al. 2018 [51]	Retro	USA	22 (13/9)	52	44	0.0	2.7	NA	NA	Charged particle radiation, linear accelerator, CyberKnife, or gamma knife	33.0	7
Hasegawa et al. 2019 [52]	Retro	Japan	189 (27/162)	59	11	83.0	1.6	2.2	20.0	Gamma knife surgery	136.0	7

**Table 2:** Subgroup analyses for obliteration rate, rehemorrhage rate, and permanent neurological deficits.

Outcomes	Factors	Groups	No. of studies	OR and 95% CI	*P* value	*I* ^2^ (%)	*P*_*Q*__statistic_	*P* value between subgroups
Obliteration rate	Study design	Prospective	2	1.18 (0.45-3.08)	0.731	0.0	0.946	0.188
		Retrospective	16	0.54 (0.41-0.71)	< 0.001	68.8	< 0.001	
	Country	Eastern	6	0.57 (0.41-0.79)	0.001	0.0	0.446	0.538
		Western	12	0.55 (0.39-0.77)	< 0.001	75.4	< 0.001	
	Sample size	≥ 100	8	0.58 (0.40-0.84)	0.004	84.3	< 0.001	0.358
		< 100	10	0.53 (0.37-0.76)	0.001	0.0	0.878	
	Mean age (years)	≥ 30	13	0.55 (0.39-0.77)	0.001	74.9	< 0.001	0.872
		< 30	5	0.64 (0.48-0.86)	0.003	0.0	0.724	
	Nidus volume (ml)	≥ 10	6	0.56 (0.34-0.91)	0.020	0.0	0.823	< 0.001
		< 10	10	0.48 (0.38-0.61)	< 0.001	39.4	0.095	
	Margin dose (Gy)	≥ 20	8	0.53 (0.39-0.72)	< 0.001	56.7	0.024	< 0.001
		< 20	8	0.58 (0.39-0.87)	0.009	53.8	0.034	
	Intervention	Linear accelerator	4	0.47 (0.30-0.73)	0.001	0.0	0.789	< 0.001
		Gamma knife surgery	9	0.49 (0.38-0.64)	< 0.001	45.4	0.066	
		Not mentioned	5	0.96 (0.79-1.17)	0.688	0.0	0.624	
	Follow-up (months)	≥ 60	9	0.59 (0.41-0.86)	0.006	80.3	< 0.001	0.036
		< 60	9	0.48 (0.37-0.63)	< 0.001	0.0	0.778	
	Study quality	High	11	0.52 (0.38-0.72)	< 0.001	77.7	< 0.001	0.492
		Low	7	0.73 (0.46-1.16)	0.182	0.0	0.618	
Rehemorrhage rate	Study design	Prospective	1	0.40 (0.04-3.86)	0.428	—	—	0.395
		Retrospective	9	1.05 (0.81-1.37)	0.708	11.1	0.343	
	Country	Eastern	3	1.86 (0.30-11.58)	0.507	51.1	0.129	0.777
		Western	7	1.06 (0.86-1.30)	0.583	0.0	0.476	
	Sample size	≥ 100	5	1.10 (0.89-1.34)	0.373	0.0	0.977	0.208
		< 100	5	0.71 (0.21-2.42)	0.583	47.8	0.105	
	Mean age (years)	≥ 30	9	1.09 (0.89-1.33)	0.418	0.0	0.565	0.085
		< 30	1	0.23 (0.04-1.33)	0.100	—	—	
	Nidus volume (ml)	≥ 10	2	1.87 (0.02-153.75)	0.780	85.2	0.009	0.881
		< 10	6	1.03 (0.72-1.49)	0.864	0.0	0.854	
	Margin dose (Gy)	≥ 20	3	1.13 (0.75-1.70)	0.569	0.0	0.882	0.833
		< 20	5	0.85 (0.38-1.92)	0.701	53.9	0.070	
	Intervention	Linear accelerator	1	0.37 (0.07-1.99)	0.247	—	—	0.464
		Gamma knife surgery	5	1.09 (0.75-1.58)	0.665	0.0	0.976	
		Not mentioned	4	0.93 (0.25-3.37)	0.909	61.1	0.052	
	Follow-up (months)	≥ 60	6	1.07 (0.68-1.69)	0.763	32.4	0.193	0.496
		< 60	4	0.91 (0.56-1.48)	0.712	0.0	0.604	
	Study quality	High	5	1.08 (0.88-1.32)	0.451	0.0	0.731	0.474
		Low	5	0.87 (0.23-3.27)	0.841	44.3	0.127	
Permanent neurological deficits	Study design	Prospective	0	—	—	—	—	—
		Retrospective	7	0.80 (0.48-1.33)	0.385	18.8	0.286	
	Country	Eastern	2	0.69 (0.13-3.67)	0.663	0.0	0.981	0.898
		Western	5	0.84 (0.44-1.59)	0.585	45.8	0.117	
	Sample size	≥ 100	5	0.89 (0.42-1.86)	0.751	41.2	0.146	0.650
		< 100	2	0.64 (0.27-1.55)	0.325	0.0	0.537	
	Mean age (years)	≥ 30	6	0.81 (0.46-1.44)	0.480	32.3	0.194	0.926
		< 30	1	0.67 (0.04-11.73)	0.784	—	—	
	Nidus volume (ml)	≥ 10	1	0.54 (0.19-1.53)	0.245	—	—	0.562
		< 10	5	0.83 (0.38-1.80)	0.631	35.9	0.182	
	Margin dose (Gy)	≥ 20	4	0.81 (0.30-2.17)	0.674	50.9	0.106	0.774
		< 20	3	0.83 (0.42-1.65)	0.591	0.0	0.550	
	Intervention	Linear accelerator	1	1.00 (0.19-5.25)	1.000	—	—	0.595
		Gamma knife surgery	5	0.72 (0.36-1.45)	0.358	37.0	0.174	
		Not mentioned	1	1.23 (0.41-3.69)	0.712	—	—	
	Follow-up (months)	≥ 60	4	1.40 (0.71-2.79)	0.330	0.0	0.732	0.023
		< 60	3	0.50 (0.28-0.89)	0.018	0.0	0.622	
	Study quality	High	6	0.82 (0.46-1.46)	0.498	32.3	0.194	0.929
		Low	1	0.70 (0.09-5.50)	0.735	—	—	

## Data Availability

All data supporting this meta-analysis are from previously reported studies and datasets, which have been cited.

## Supplementary Materials

Supplementary Materials Supplement 1 Sensitivity Analysis. Figure S1: sensitivity analysis for SRS following embolization versus SRS alone on the risk of obliteration rate. Figure S2: sensitivity analysis for SRS following by prior embolization versus SRS alone on the risk of rehemorrhage. Figure S3: sensitivity analysis for SRS following embolization versus SRS alone on the risk of permanent neurological deficits. Supplement 2 publication bias. Figure S1: publication bias for the obliteration rate. Figure S2: publication bias for the rehemorrhage rate. Figure S3: publication bias for permanent neurological deficits.
