# Supplementary material for: Efficacy and Safety of Combined Endovascular Embolization and Stereotactic Radiosurgery for Patients with Intracranial Arteriovenous Malformations: A Systematic Review and Meta-Analysis
Source: Biomed Res Int. 2021 Apr 14;2021:6686167. doi: 10.1155/2021/6686167 (PMC8060080; doi:10.1155/2021/6686167)
Supplement: Supplementary Materials — Supplement 1 Sensitivity Analysis. Figure S1: sensitivity analysis for SRS following embolization versus SRS alone on the risk of obliteration rate. Figure S2: sensitivity analysis for SRS following by prior embolization versus SRS alone on the risk of rehemorrhage. Figure S3: sensitivity analysis for SRS following embolization versus SRS alone on the risk of permanent neurological deficits. Supplement 2 publication bias. Figure S1: publication bias for the obliteration rate. Figure S2: publication bias for the rehemorrhage rate. Figure S3: publication bias for permanent neurological deficits. [file 6686167.f1.docx]

Supplement 1


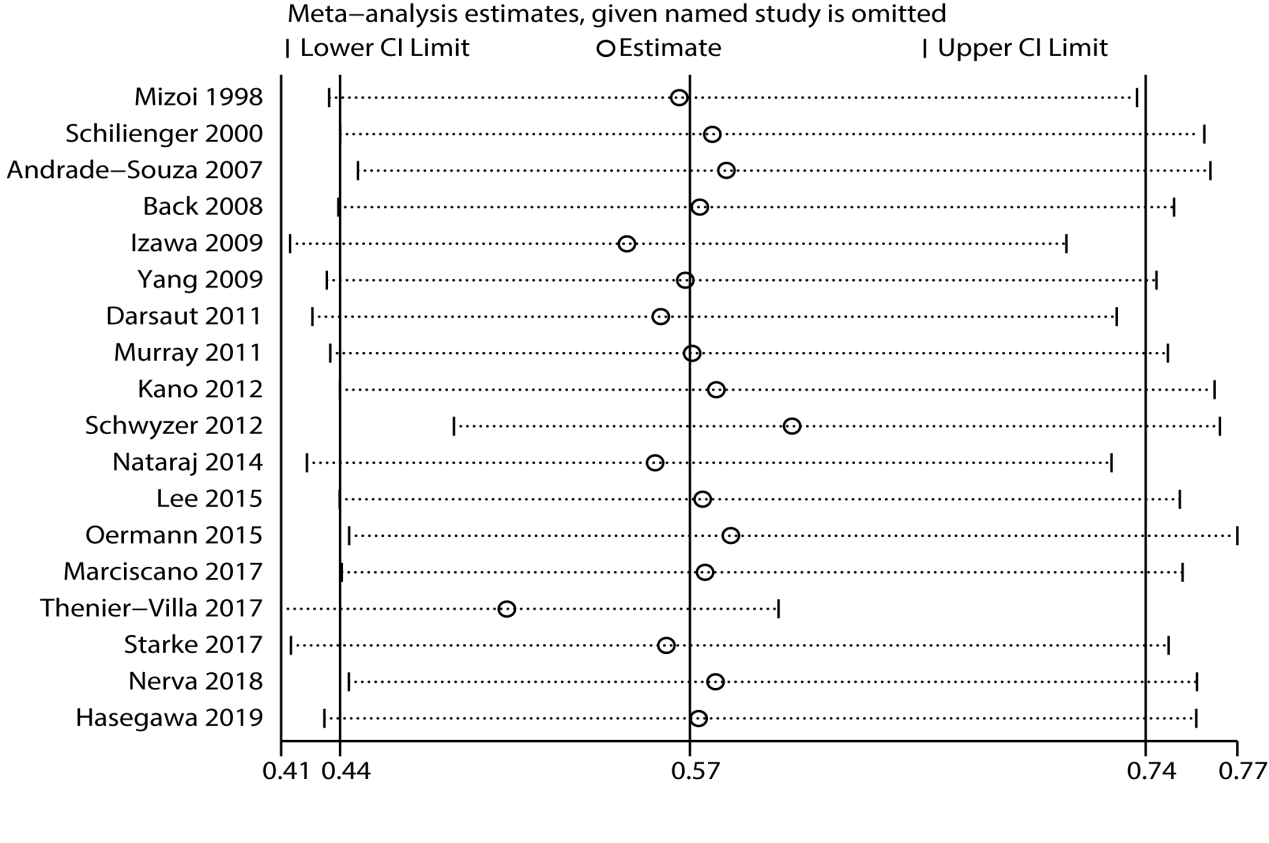


Figure S1. Sensitivity analysis for SRS following embolization versus SRS alone on the risk of obliteration rate


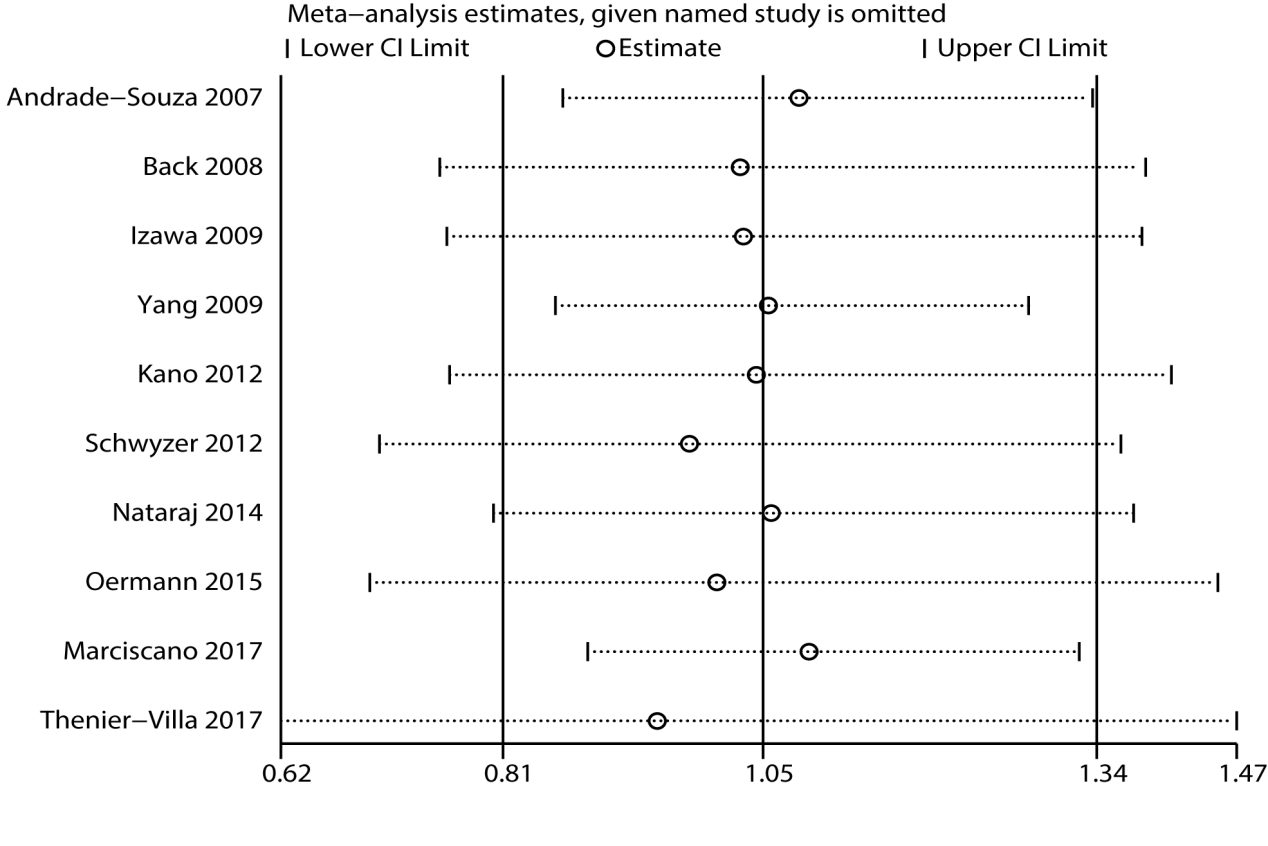


Figure S2. Sensitivity analysis for SRS following by prior embolization versus SRS alone on the risk of rehemorrhage


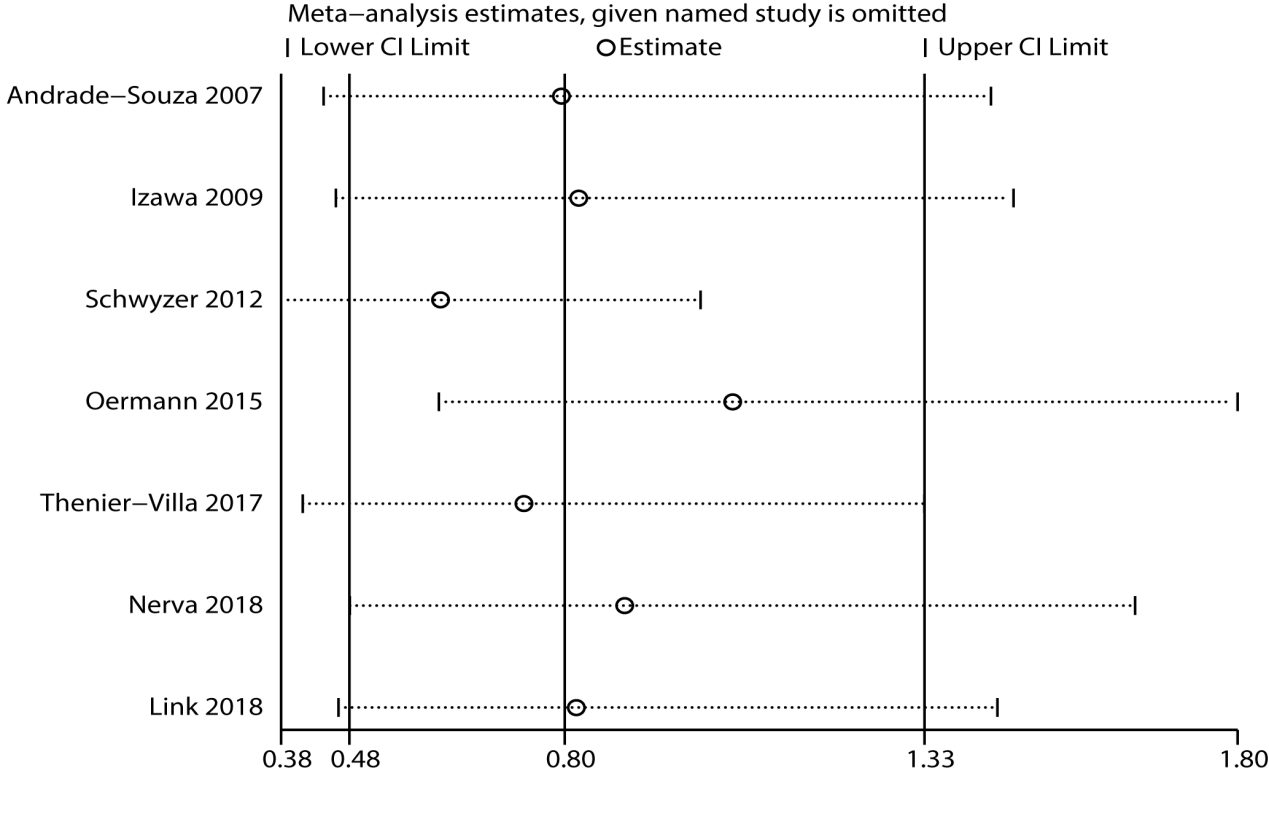


Figure S3. Sensitivity analysis for SRS following embolization versus SRS alone on the risk of permanent neurological deficits

Supplement 2


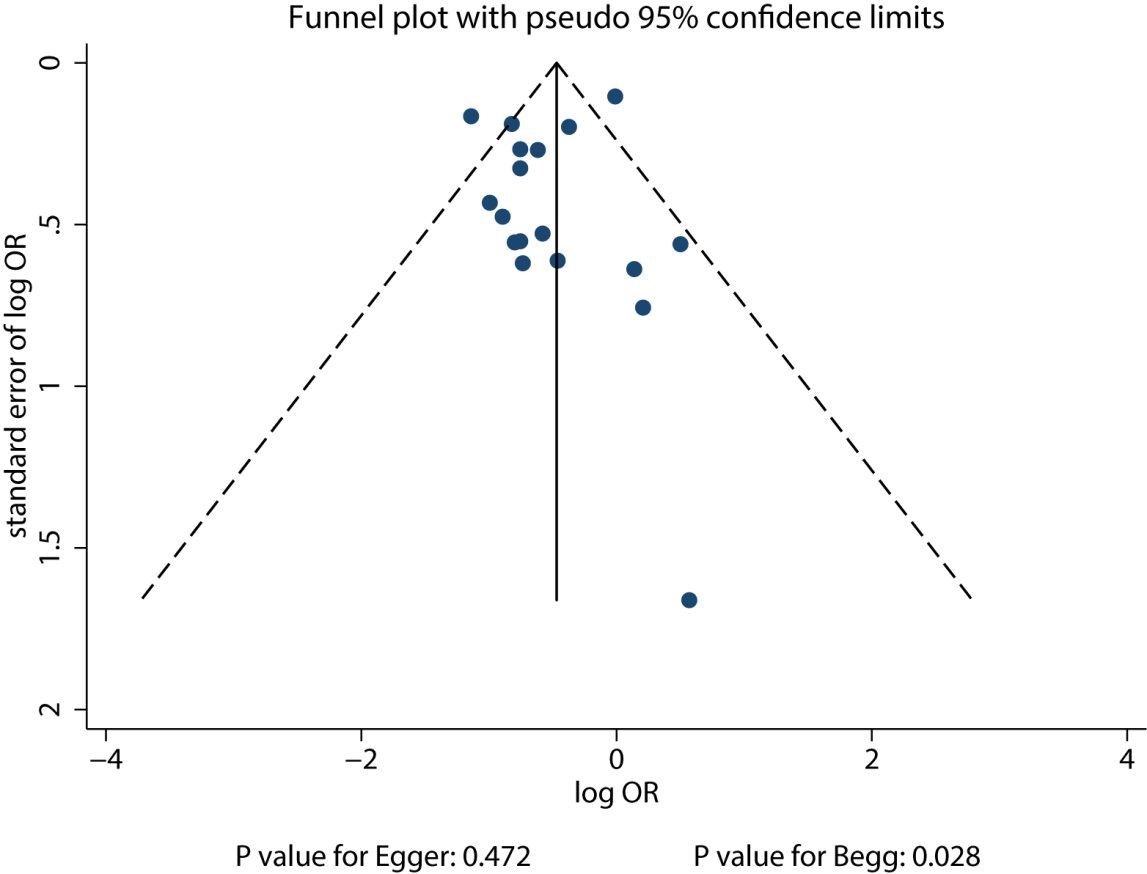


Figure S1. Publication bias for obliteration rate


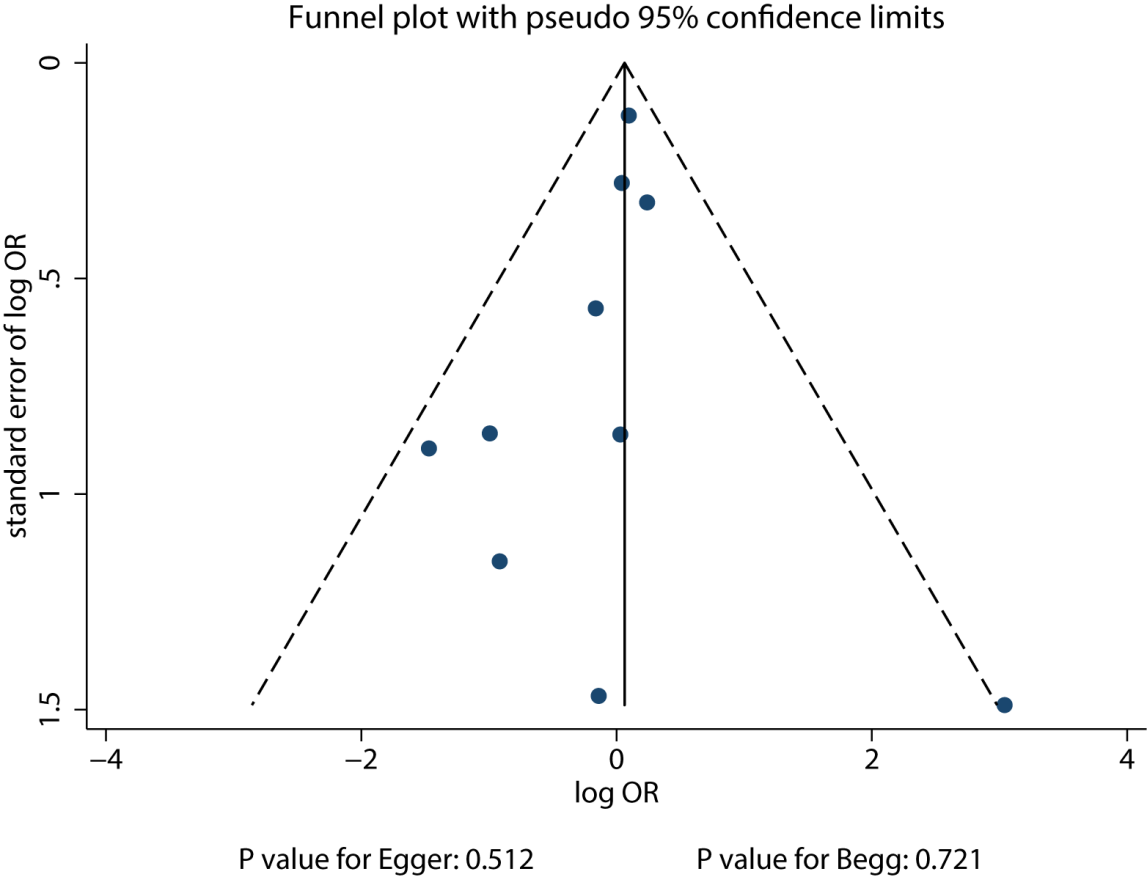


Figure S2. Publication bias for rehemorrhage rate


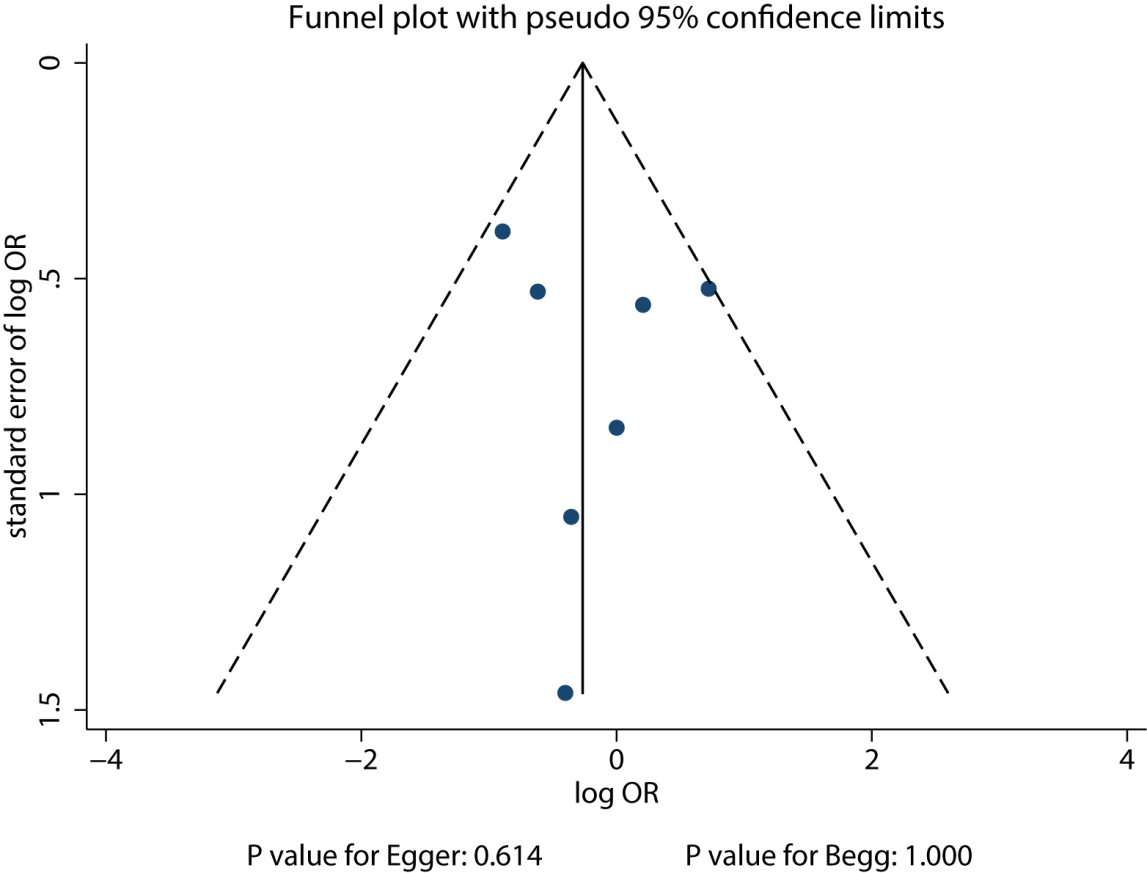


Figure S3. Publication bias for permanent neurological deficits
